# Synthesis, Modification and Biological Activity of Diosgenyl β-d-Glycosaminosides: An Overview

**DOI:** 10.3390/molecules25225433

**Published:** 2020-11-20

**Authors:** Daria Grzywacz, Beata Liberek, Henryk Myszka

**Affiliations:** Laboratory of Glycochemistry, Faculty of Chemistry, University of Gdańsk, Wita Stwosza 63, 80-308 Gdańsk, Poland; beata.liberek@ug.edu.pl (B.L.); henryk.myszka@ug.edu.pl (H.M.)

**Keywords:** steroid saponin, diosgenin glycosides, diosgenyl β-d-glucosaminoside, diosgenyl β-d-galactosaminoside, amine group modifications, antimicrobial activity, anti-cancer activity, hemolytic activity

## Abstract

Saponins are a structurally diverse class of natural glycosides that possess a broad spectrum of biological activities. They are composed of hydrophilic carbohydrate moiety and hydrophobic triterpenoid or steroid aglycon. Naturally occurring diosgenyl glycosides are the most abundant steroid saponins, and many of them exhibit various pharmacological properties. Herein, we present an overview of semisynthetic saponins syntheses–diosgenyl β-d-glycosaminosides (d-gluco and d-galacto). These glycosides possess a 2-amino group, which creates great possibilities for further modifications. A wide group of glycosyl donors, different *N*-protecting groups and various reaction conditions used for their synthesis are presented. In addition, this paper demonstrates the possibilities of chemical modifications of diosgenyl β-d-glycosaminosides, associated with functionalisation of the amino group. These provide *N*-acyl, *N*-alkyl, *N,N*-dialkyl, *N*-cinnamoyl, 2-ureido and 2-thiosemicarbazonyl derivatives of diosgenyl β-d-glycosaminosides, for which the results of biological activity tests (antifungal, antibacterial, anti-cancer and hemolytic) are presented.

## 1. Introduction

The aim of this review is to provide information on the methods of synthesis and biological activity of diosgenyl β-d-glycosaminosides and their derivatives. These are semisynthetic saponins with proven antimicrobial and antitumor activity. This makes them very promising candidates for use as an antifungal or antibacterial drug. A significant advantage of these compounds is that they are not toxic. The toxicity of saponins is a particular limitation in the clinical application of this group of compounds.

## 2. Saponins, Their Occurrence, Properties and Structure

Saponins are a structurally diverse group of glycosides and are widely distributed in nature. Although these compounds are typical for plants [1,2], they have also been isolated from animals [3,4].

Saponins have the characteristic ability to reduce the surface tension of aqueous solutions and maintain a stable foam [5]. Therefore, they are useful in the production of emulsions and cleaning agents [5]. Most of them have been extracted from herbal preparations used in folk medicine, especially in Asian countries. In the form of herbal extracts, ointments, and various types of infusions, they are used as anti-malarial drugs, antidotes against snake and insect venoms, and as antiseptics, bactericides and antivirals [6]. Saponins also present interesting pharmacological properties, such as anti-diabetic [7,8,9], anti-cancer [10,11,12] and anti-inflammatory [13,14,15,16], and specific physiological properties—they change the structure of cell membranes, making them more permeable to compounds [17]. Saponins can also impair the digestion of intestinal proteins and the absorption of vitamins and minerals [18].

Saponins are composed of hydrophilic carbohydrate moiety and hydrophobic sapogenin. Due to their structure classic methods of saponin isolation, such as: solvent extraction, column chromatography and preparative TLC, are in many cases insufficient to isolate single saponins from plant material. Therefore, various, more modern, often combined separation techniques are used. Depending on the type of plant material, the following methods of extracting saponins can be distinguished: microwave-assisted solvent extraction (MAE); ultrasound-assisted solvent extraction (UAE); solid phase extraction (SPE); preparative column chromatography (CC); high-performance liquid chromatography (HPLC) coupled with other techniques [19,20].

**Figure 1:**
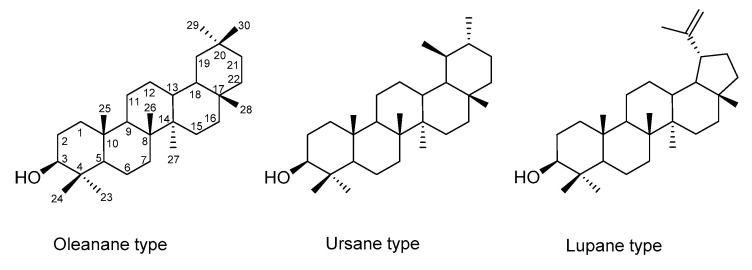
Chemical structures of triterpenoid sapogenins and the numbering system of carbon atoms.

The division of saponins depends mainly on the type of sapogenin. Triterpenoid and steroid saponins are one of the most important families of these plant secondary metabolites. Triterpenoid aglycones have the most varied structures among all types of sapogenins and dominate in the plant kingdom [21]. Their aglycon usually contains 30 carbon atoms and comprises five six-carbon rings (oleanane and ursane type; Figure 1) or four six- and one five-carbon ring (lupane type, Figure 1). A common feature of this type of sapogenins is the arrangement of the methyl substituents. Groups at the C-4, C-8 and C-10 atoms occupy the β position, whereas groups at the C-4 and C-14 atoms occupy the α position. The presence of a hydroxyl group at the secondary carbon atom (C-3) is also typical for triterpenoid sapogenins.

**Figure 2:**
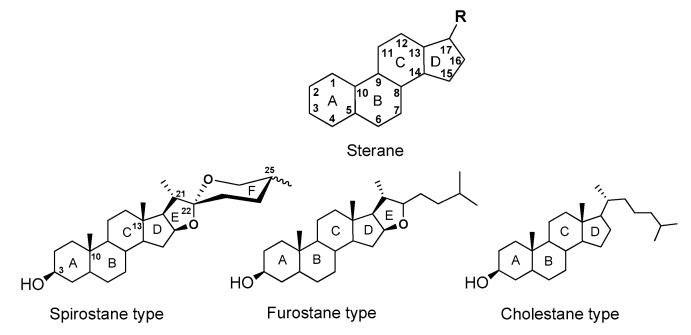
Chemical structures of steroidal sapogenins and the numbering system of carbon atoms.

The basic aglycone of steroid saponins contains 17 carbon atoms and is based on the sterane skeleton (1,2-cyclopentanoperhydrophenantrene), which comprises three six- and one five-carbon ring (Figure 2). Steroid sapogenins differ in the structure of the substituent located at the C-17 atom, which could be an additional heterocyclic ring or an aliphatic chain. Due to the nature of this substituent, steroid saponins are divided into three types: spirostane, furostane and cholestane (Figure 2) [22]. A common feature in the structure of steroid sapogenins is the arrangement of the methyl substituents located at the C-10 and C-13 atoms, which occupy the β position, whereas these occupy the α position at the C-21 atom. The presence of a hydroxyl group at the C-3 atom is also typical for steroidal saponins. Individual sapogenins differ in the presence of double bonds (e.g., C5 = C6), the configuration of the methyl group at the C-25 atom in spirostane saponins (25*R* or 25*S*) and sometimes the presence of additional functional groups (e.g., -OH).

The structural diversity of saponins lies also in the hydrophilic fragment, which usually comprises one or more sugar units. Analysis of the structure–activity relationship (SAR) has proven that the sugar portion plays an important role in the biological activity and might be the key pharmacophore for saponins’ anti-cancer activities [23]. The most common saccharides found in saponins are: β-d-glucopyranose, β-d-galactopyranose, α-l-rhamnopyranose and β-d-xylopyra- nose; di-tri- and tetrasaccharides also occur. *N*-Acetyl-d-glucosamine residue sometimes is attached as the first sugar to the triterpenoid sapogenin [24]; however, in spirostane saponins d-glucose, and less frequently, d-galactose, are usually directly attached to sapogenin. Natural steroidal saponins that contain an amino sugar fragment are very rare.

## 3. Diosgenyl Saponins

**Figure 3:**
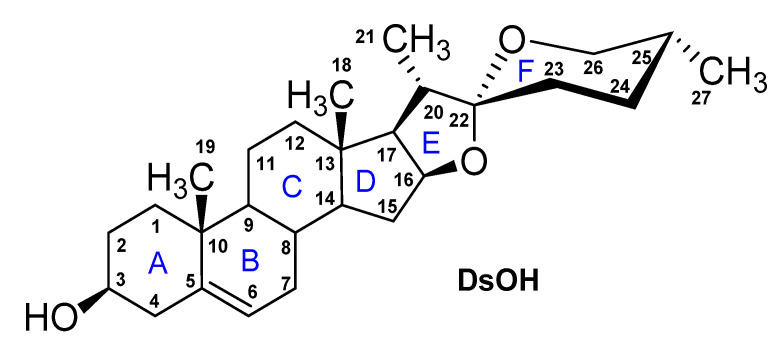
The structure of diosgenin (DsOH) with the numbering system of carbon atoms.

Diosgenin ((25*R*)-spirost-5-en-3β-ol; DsOH; Figure 3) is a spirostane, and it has a very high structural similarity to steroid hormones. Therefore, it is a valuable and often used precursor in the synthesis of hormones and corticosteroids, including cortisone, pregnenolone and progesterone, on an industrial scale [25]. It is known for its anti-inflammatory and antioxidant properties and can also be used in the treatment of allergic and metabolic diseases (hypercholesterolemia, dyslipidaemia, diabetes and obesity), as well as for menopause symptoms and skin aging [26].

Diosgenin in combination with carbohydrate forms diosgenyl glycosides. In these natural compounds, d-glucose is usually the saccharide directly attached to sapogenin. However, combinations of diosgenin with other sugars have also been found: d-galactose (e.g., smilacinoside A, funcioside B or indioside E) and l-arabinose (e.g., conwallasaponin E and polyphilin F). Diosgenyl glycosides are the most abundant and, from the pharmaceutical point of view, the most explored natural steroid saponins. They occur mainly in the family of fungus plants (*Dioscoreaceae*), as well as in some species of solanaceae (*Solanaceae*), bean plants (*Fabaceae*) and fenugreek (*Trigonella*) [26]. Many of them exhibit antifungal [27], anti-thrombotic [28], antivirial, antioxidative and tissue-protective properties [29]. Diosgenyl glycosides also exerted an antitumor effect by inducing apoptosis in cancer cells and have great potential to be explored for cancer treatment [30,31,32].

Diosgenin saponins are commonly present in Chinese herbal preparations used in traditional folk medicine. The turning point in the research on this saponin was the discovery of the anti-cancer properties of the Chinese preparation ‘Yunnan Baiyao’ in the 1960s and the isolation of active substances with it [33]. Since then, research on methods of diosgenin saponins synthesis and their activity has been intensified. In the following years, numerous studies of representatives this class of compounds were carried out to find compounds useful in the fight against cancer cells and in therapies of diverse clinical disorders. Among the most explored compounds are dioscin (^3^*O*-{α-l-Rha-(1→4)-[α-l-Rha-(1→2)]-β-d-Glc}-diosgenin), gracillin (^3^*O*-{α-d-Glc-(1→3)-[α-l-Rha-(1→2)]-β-d-Glc}-diosgenin) and their analogs [34,35,36].

## 4. d-Glycosaminosides of Diosgenin

Given that steroid–carbohydrate conjugates exhibit a broad spectrum of biological properties, research is ongoing not only to isolate these compounds from plant materials but also to improve the methods of their synthesis. The latter endeavour offers enormous opportunities to obtain non-naturally occurring compounds. Their design is based on the leading structure of a biologically active substance with confirmed activity. After finding that the compound exhibits biological activity, it is often chemically modified to improve its properties (physicochemical or biological) or to eliminate undesirable properties, such as high toxicity or poor solubility.

Diosgenyl d-glycosaminosides, which contain 2-amino-2-deoxy sugar residue in the carbohydrate moiety, have not yet been isolated from natural sources. Typically, d-glucose is linked to diosgenin in naturally occurring saponins. Replacing d-glucose with d-glucosamine or d-galactosamine is a promising modification; the presence of an amino group creates great opportunities for further modifications of these compounds. Amino sugars are of great biological importance and are commonly found as a component of natural oligo- and polysaccharides, glycoproteins, and can significantly increase the bioactivity of compounds. The formation of a glycosidic bond with them presents many problems, and much effort has been put into developing the most convenient conditions for such synthesis [37]. This review presents various synthetic approaches for obtaining diosgenyl β-d-glycosaminosides (different glycosyl donors, different protective groups of the amino function, different solvents used for the reaction, etc.). Moreover, the chemical modifications of the obtained diosgenyl β-d-glycosaminosides that lead to *N*-acyl, *N*-alkyl, *N,N*-dialkyl, *N*-cinnamoyl, 2-ureido and 2-thiosemicarbazyl derivatives, as well as the results of biological activity tests (antifungal, antibacterial, anti-cancer and hemolytic) of these new saponins, are presented.

### 4.1. Methods of Synthesis

The general strategy for the synthesis of diosgenyl 2-amino-2-deoxy-β-d-glycopyranoside (diosgenyl β-d-glucosaminoside, DsO-β-d-Glc-NH_2_) relies on the preparation of an appropriately protected glycosyl donor, next, a coupling of the donor with diosgenin and finally, the deprotection of the amine and hydroxyl groups of the obtained diosgenyl glycoside. A properly protected amino group located at the C-2 atom of the glycosyl donor can play a key role in a glycosidic coupling, e.g., amide-type groups participate in the process of bond forming as the *neighbouring group*, which favours the formation of a 1,2-*trans*-glycosidic bond [38]. Therefore, among other things, various *N*-protecting groups were developed for the synthesis of diosgenyl–carbohydrate conjugates.

**Scheme 1:**
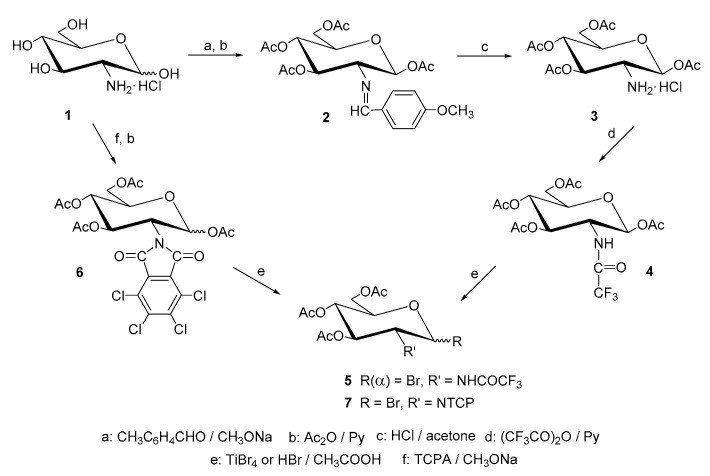
Preparation of bromides **5** and **7** with the trifluoroacetyl (TFAc) and tetrachlorophthatloyl (TCP), respectively, *N*-protecting groups.

The first synthesis of diosgenyl 2-amino-2-deoxy-β-d-glucopyranoside was presented by Bednarczyk et al. in 2000 [39]. Two differently *N*-protected bromides (**5**,**7**) were used as the glycosyl donors (Scheme 1). The first one was 3,4,6-tri-*O-*acetyl-2-deoxy-2-trifluoroacetamido-α-d-glucopyranosyl bromide (**5**), which was syntesised via acetate **3** [40]. A trifluoroacetyl (TFAc) group was introduced on the free NH_2_ function (**4**), formed by removing the imino group from **2** [41]. The final step was bromination of **4** with TiBr_4_. The second bromide-3,4,6-tri-*O*-acetyl-2-deoxy-2-(3,4,5,6-tetrachlorophthalimido)-α,β-d-glucopyranosyl bromide (**7**) differs from the former one (**5**) in the amine group protection. In this case, a tetrachlorophthaloyl (TCP)-protecting group was used to block the amine function. From the *N*-TCP-protected acetate **6**, in reaction with TiBr_4_, bromide **7** was synthesised. This time, the first was acylation of the amine function in hydrochloride **1** with tetrachlorophthalic anhydride (TCPA), followed by acetylation of the *N*-protected product with acetic anhydride in pyridine. This approach afforded compound **6** (α/β), and from this, the mixture of the anomeric bromides **7** was obtained. Furthermore, bromides **5** and **7** were also synthesised with the use of HBr in acetic acid [42]. The yields of both manners of bromination are similar. 

When comparing these approaches, one may see that the overall efficiency of the acetate **4** and **6** synthesis is higher when the acetylation reaction is carried out first, followed by amino function protection. In this approach, only the β-anomer of **4** (81%) is obtained. However, when the amine function is first protected and followed by acetylation, the acetate **6** (62%) is a mixture of α- and β-anomers, with clear dominance of the latter. This observation depends on the protecting groups used and has also been confirmed for different types of amino protecting groups (e.g., 2,2,2-trichoroetoxycarbonyl or phthaloyl groups) [43].

**Scheme 2:**
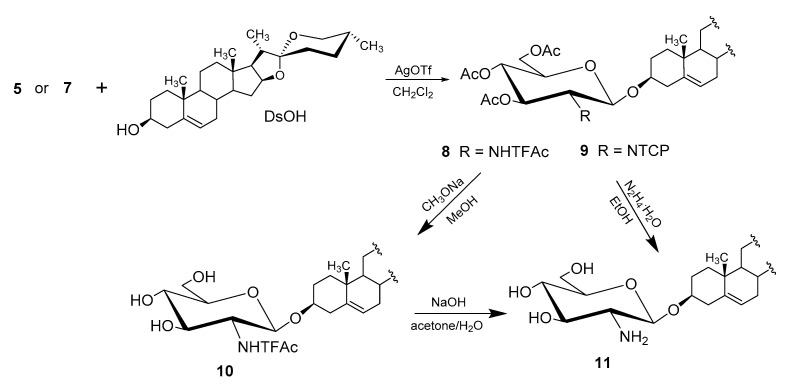
Glycosylation of diosgenin with bromides **5** and **7**.

Condensations of the obtained glycosyl donors (bromides **5** and **7**) with diosgenin were carried out according to the modified Koenigs–Knorr method [44] that employed silver triflate (AgOTf) as the reaction promoter, in combination with powdered 4 Å molecular sieves in anhydrous dichloromethane (CH_2_Cl_2_) under an inert gas, namely nitrogen (Scheme 2) [39,41]. In this way, saponins **8** and **9** were obtained. The reaction of **8** with 1 M sodium methoxide in methanol yielded the partially deprotected diosgenyl 2-deoxy-2-trifluoroacetamido-β-d-glucopyranoside (**10**). The treatment of **10** with 1 M aqueous sodium hydroxide in acetone, followed by neutralisation, yielded the fully deprotected diosgenyl 2-amino-2-deoxy-β-d-glucopyranoside (**11**), which was isolated as the hydrochloride (**11^.^**HCl). In turn, the full *O*- and *N*-deprotection of **9**, in the presence of hydrazine hydrate in ethanol, was described [43]. This also led to the fully deprotected **11**. Under such conditions, glycosylations were efficient (~65%) and stereoselective, resulting only in β-glycosides. Importantly, this configuration also occurs in the natural diosgenyl glycosides. 

**Figure 4:**
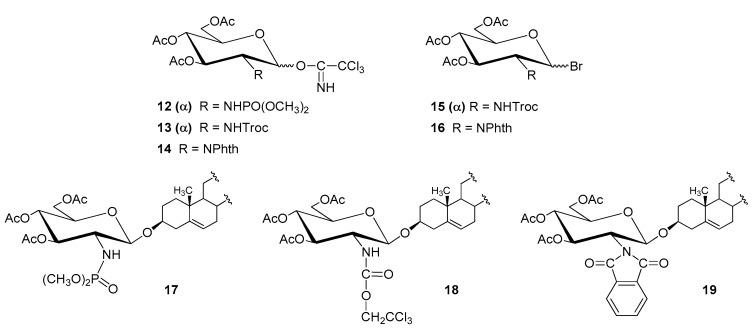
Glycosyl donors: trichloroacetimidates (**12**–**14**) and bromides (**15**,**16**) with different protecting groups at the amine function and examples of diosgenyl β-d-glucosaminosides synthesised with them (**17**–**19**).

In 2007, Yu and co-workers described another way of synthesis protected diosgenyl β-d-glucosaminoside using a different group of the glycosyl donors—trichloroacetimidate (TCAI), namely 3,4,6-tri-*O*-acetyl-2-deoxy-2-*N*-dimethylphosphoryl-α-d-glucopyranosyl trichloroacetimidate (**12**, Figure 4) [45]. Synthesis of **12** involves treatment of acetate **3** with diphenyl chlorophosphate in the presence of 4-dimethylaminopyridine (DMAP) and Et_3_N, which gave *N*-diphenylphosphoryl (DPP)-glucosamine derivative. Transesterification of DPP into a dimethylphosphoryl group (DMP) and removal of the anomeric *O*-acetyl group, followed by reaction with trichloroacetonitrile (CCl_3_CN), resulted in a new glycosyl donor (**12**). Its reaction with diosgenin in the presence of trimethylsilyl trifluoromethanesulfonate (TMSOTf) gave saponin **17**, with a 92% yield. From glycoside **17,** *O*-acetyl groups and DMP were finally removed in the presence of NaOH or hydrazine [45].

In the subsequent years, other glycosyl donors were tested for the synthesis of diosgenyl glucosaminosides. Kaskiw et al. used 3,4,6-tri-*O*-acetyl-2-deoxy-2-(2′,2′,2′-trichloroethoxycarbonyl- amino)-α-d-glucopyranosyl trichloroacetimidate (**13**) [46]. The 2,2,2-trichoroetoxycarbonyl group (Troc) is stable under a wide range of standard conditions used in the synthesis of glucosamine derivatives. Additionally, it belongs to potential participating groups, which promote the formation of 1,2-*trans*-glycosides. The authors obtained saponin **18** (98%) in the reaction of **13** with diosgenin in the presence of TMSOTf as the catalyst (Figure 4). 

**Scheme 3:**
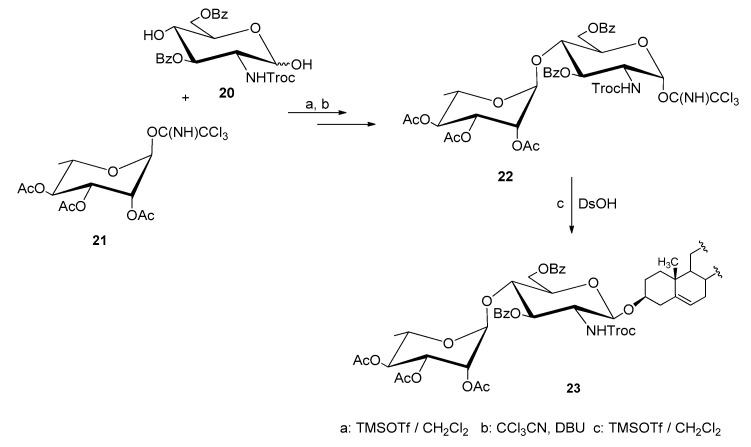
Glycosylation of diosgenin with *N*-Troc-amino disaccharide (**22**).

The Kaskiw’s group also used the glycosyl trichloroacetimidate donor with the Troc-protecting group on an amine function in the synthesis of protected diosgenyl amino disaccharide (**23**) [47]. The attached disaccharide comprises benzoylated d-glucoaminopyranose with a Troc-protecting group (**20**) and acetylated l-rhamnopyranose (**21**) (Scheme 3). The glycosylation of diosgenin was performed with the respective trichloroacetimidate (**22**) in the presence of TMSOTf in an 80% yield. The resulting glycoside was further transformed, and the protecting groups were subsequently removed by treatment with sodium methoxide in methanol.

In turn, Fernandez-Herrera et al. proposed per-*O*-acetylated 1-*O*-trichloroacetimidate, with phthaloyl protection (Phth) of the amine function (**14**), as a suitable donor for diosgenin glycosylation (Figure 4). They obtained only β-anomer of saponin **19** in a glycosylation reaction promoted by TMSOTf; the yield was 96% [48]. Tan’s research group also used a similar procedure to obtain **19**, but with a slightly worse yield (80%) [49]. Saponins **18** and **19** were also synthesised in the reactions of diosgenin with bromides **15** and **16**, respectively (Figure 4) [50]. The authors obtained only the α anomer of **15** and a mixture of anomers α + β in a case of **16**. These bromides were later used without further purification in the coupling reaction with diosgenin in the presence of AgOTf. The reaction yields were 98% and 90%, respectively.

**Figure 5:**
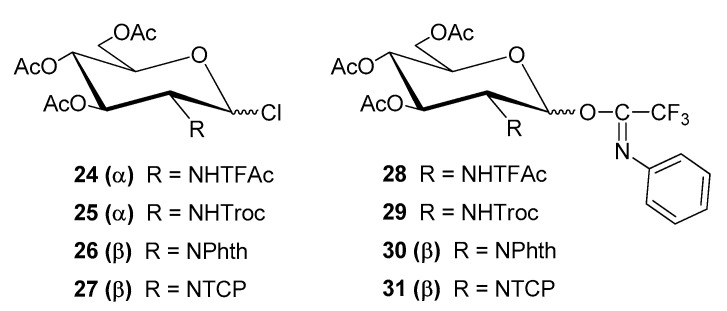
Glycosyl chlorides (**24**–**27**) and (*N*-phenyl)trifluoroacetimidates (**28**–**31**) with different protecting groups at the 2-amino function.

In addition to the above-described and commonly used d-glucosaminosyl donors, the less frequently used d-glucosaminosyl chlorides (**24**–**27**) and (*N*-phenyl)trifluoroacetimidates (PTFAI, **28**–**31**) are also noteworthy (Figure 5) [43]. Although glycosyl chlorides are less reactive than corresponding bromides, they are more stable and were used for the synthesis of diosgenyl glycosides.

There are several ways that a chlorine atom can be introduced on an anomeric carbon atom in *N*-protected and per-*O*-acetylated d-glucosamine. One of the often-used chlorinating agents is 1,1-dichloromethyl methyl ether in the presence of ZnCl_2_ or BF_3_·H_2_O [51]. This reagent was successfully used by Bednarczyk et al. to synthesise chlorides **24**–**27** [43]. Chlorides with the 2-NHTFAc (**24**) or 2-NHTroc (**25**) groups were solely α anomers, whereas those with imide-type moieties (2-NPhth **26** and 2-NTCP **27**) have the β configuration on the anomeric carbon atom. Glycosyl chlorides (**24**–**27**) were used in coupling reactions with diosgenin, carried out in CH_2_Cl_2_ or in its mixture with Et_2_O, in the presence of AgOTf as a reaction promoter. The fully protected diosgenyl β-d-glucosaminosides (**8**,**9**,**18**,**19**) were obtained with a yield of 69–99% [43].

The use of (*N*-phenyl) trifluoroacetimidates (PTFAI) instead of trichloroacetimidates (TCAI) as glycosyl donors is an alternative method for the synthesis of glycosides [52,53], including diosgenyl saponins. The synthesis of 1-*O*-(*N*-phenyl)trifluoroacetimidates (**28**–**31**, Figure 5) from the respective sugar acetates with four different 2-*N*-protecting groups (TFAc, Troc, Phth and TCP) requires the selective removal of the acetyl group from the anomeric carbon, typically with ethylenediamine in a mixture of acetic acid in THF, followed by reaction with (*N*-phenyl)trifluoroacetate imidoyl chloride. This approach was ineffective in the case of d-glucosaminopyranose acetate with the TCP-protecting group (**6**); therefore, its bromide or chloride was hydrolysed in reaction with Ag_2_CO_3_ in the acetone and H_2_O mixture. It was then reacted with freshly prepared (*N*-phenyl)trifluoroacetate imidoyl chloride [43]. Such synthesised glycosyl donors in the reaction with diosgenin in the presence of TMSOTf led to the expected fully protected β-glycosides (**8**,**9**,**18**,**19**) obtained in yields of 52–85%.

**Scheme 4:**
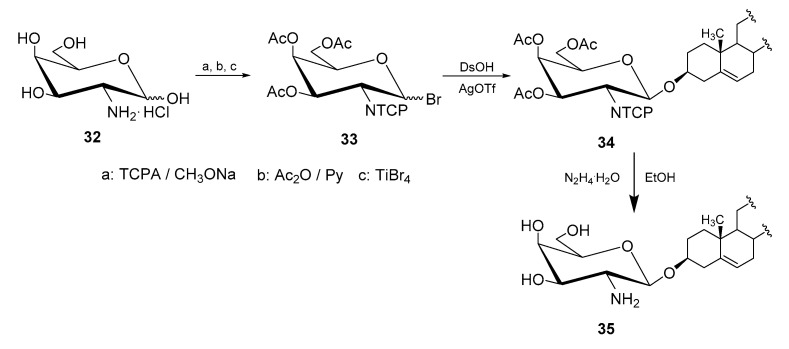
Synthesis of diosgenyl β-d-galactosaminoside (**35**).

In addition to d-glucosamine, 2-amino-2-deoxy-d-galactopyranose (d-galactosamine) was glycosidically attached to diosgenin [54]. Natural diosgenyl d-galactosides have been much less isolated from plants than the corresponding d-glucosides. Similarly to diosgenyl glucosaminosides, spirostane saponins that contain a d-galactosamine in carbohydrate portion are not found in nature. To synthesise diosgenyl β-d-galactosaminosides (**35**), analogous reactions were performed, such as those described for the d-glucosamine series (Scheme 4). Thus, to obtain bromide **33**, d-galactosamine hydrochloride (**32**) was used. It was first acylated with tetrachlorophthalic anhydride (TCPA), followed by acetylation with acetic anhydride in pyridine. Then, the obtained anomeric mixture of the product was brominated with TiBr_4_, which led to an anomeric mixture of bromides (**33**), with a clear predominance of the β anomer (α:β = 1:4). Due to the high reactivity of bromides, this donor was immediately used in the condensation reaction with diosgenin in CH_2_Cl_2_, in the presence of AgOTf as the reaction promoter. There was an 80% yield of synthetic protected diosgenyl β-d-galactosaminoside (**34**) [54]. Deprotection of the *O*-acetyl groups and NTCP group of **34** was achieved by using 98% hydrazine hydrate in EtOH and yielded diosgenyl 2-amino-2-deoxy-β-d-galactopyranoside (**35**), which was converted into hydrochloride **35^.^**HCl.

Glycosylation of diosgenin with d-glycosamine derivatives mainly proceeds according to the S_N_1 and/or S_N_2, mechanism, usually with the contribution of protected 2-amino groups (NHTFAc, NHTroc, NPhth and NTCP). The presence of these groups promotes the formation of 1,2-*trans* glycosides, which in the case of d-glucosamine and d-galactosamine means formation of β-configurated glycosides.

The type of promoter used (heavy metal salt or TMSOTf), as well as the solvent and temperature, are also significant. Table 1 summarizes the most useful procedures of providing diosgenyl β-d-glycosaminosides. The type of glycosyl donor (bromides, chlorides and imidates) and the order of reagent addition has a noticeable influence on the efficiency of the reaction. For the glycosidation reaction, two procedures are recognized: *normal* and *reverse*. In the *normal* procedure, the promoter (AgOTf or TMSOTf) is added as the last to the solution containing the glycosyl donor and acceptor, whereas in the *reverse* procedure, the glycosyl donor is added to the mixture of the promoter and glycosyl acceptor [55].

The choice of glycosylation procedure should also consider the orthogonality of the protecting groups, and thus the possibility of removing them independently. This factor is especially important when there is a planned modification of the obtained glycoside, e.g., by attaching further saccharide units or functionalising the amino group. 

*O*-Acetyl groups used in the above presented syntheses could be easily removed under basic conditions (typically with sodium methoxide in methanol). It is possible to remove only *O*-acetyl groups while the amino-protecting groups remain intact (**8**→**10**, Scheme 2). This approach is important if further attachment of the sugar units to such saponin is planned. To remove the TFAc amino-protecting group, a solution of NaOH in an acetone–water mixture is typically used (**10**→**11**, Scheme 2). Under these conditions, the *O*-acetyl groups could be also removed [57]. Likewise, the *O*-acetyl groups, the TCP- and TFAc-protecting groups can be removed simultaneously under weakly basic conditions, e.g., with hydrazine hydrate in EtOH, at the reflux temperature (**9**→**11** and **34**→**35**). If diethylamine is used instead of hydrazine hydrate, an *N*-acetyl derivative of saponin is often formed as a by-product [57]. Removal of the phthaloyl group requires a high temperature and quite a long reaction time. This reaction usually uses hydrazine hydrate or *n*-butylamine in ethanol (**19**→**11**) [58]. It is worth adding that the order in which the protecting groups are removed must be taken into account. Generally, *O*-deacetylation should be done first, otherwise *O*→*N* acetyl migration may occur.

**Scheme 5:**
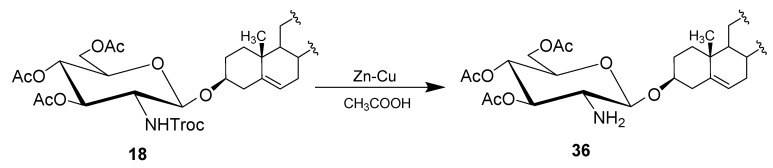
Removal of Troc protecting group from amino function.

Sometimes, to functionalise an amino group, it is necessary to remove only the protecting group from the amine function, leaving the acetyl groups on the hydroxyl functions. In that case, it is preferable to use a trichloroethoxycarbonyl group, which could be selectively removed under reductive β-elimination with zinc dust or zinc-copper powder in acetic acid (**18** → **36**, Scheme 5) [43,46].

### 4.2. Chemical Modification

Difficulties associated with the isolation and purification of saponins from natural sources force its synthesis. In addition, chemical modifications create opportunities to obtain new compounds, often with more favourable therapeutic properties compared to naturally occurring or reference substances. Saponins, especially diosgenyl glycosides, are attractive candidates for new drug design and development due to their valuable properties: anti-inflammatory [13,14], antimicrobial [27], anti-coagulant [28], anti-cancer [30,31,32] and gelling [59].

Chemical modifications of diosgenyl saponins mainly concern the sugar part. The changes most often involve attachment of other sugar residues or extension of the sugar chain and introduction of various functional groups or substituents. Modifications of aglycon have also been described in the literature [60].

Research related to the chemical modification of diosgenyl β-d-glycosaminosides is mainly associated with the functionalisation of the amino group. Thus, the following sections present the procedures for modifying the -NH_2_ group and the results of studies on the biological activity of the most interesting derivatives of diosgenyl glycosaminosides. 

#### 4.2.1. *N*-alkyl and *N*,*N*-dialkyl Derivatives

A series of *N*-alkyl and *N,N*-dialkyl derivatives of diosgenyl 2-amino-2-deoxy-β-d-gluco- and d-galactopyranosides have been synthesised [50,54]. The synthesis of these compounds used a method of reductive alkylation of amines [61]. *N*-Monoalkyl derivatives were obtained by treatment of the primary amine group in diosgenyl β-d-glycosaminoside with an appropriate aldehyde (*R*-CHO), followed by reduction in the resulting imine with sodium cyanoborohydride (NaBH_3_CN).

The *N*-alkyl derivatives, as the secondary amines, can react under the same conditions with another aldehyde molecule to form an enamine, from which the *N,N*-dialkyl derivative is obtained after reduction. Such alkylation reactions are not selective and usually provide mixtures of mono- and dialkylated products, which should be separated by column chromatography. 

**Figure 6:**
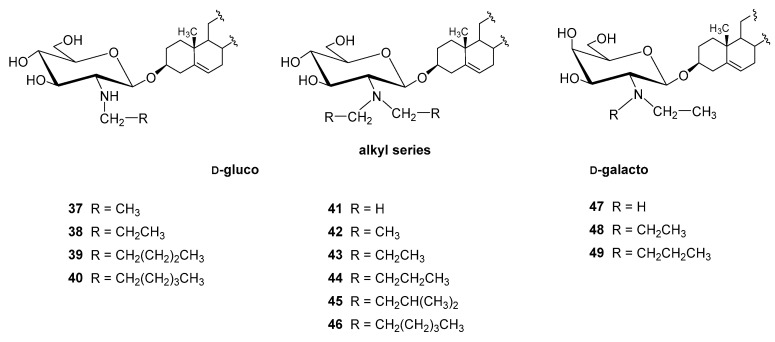
*N*-Alkyl and *N,N*-dialkyl derivatives of diosgenyl β-d-gluco-(**37**–**46**) and β-d-galactosaminosides (**47**–**49**).

Using reductive alkylation of diosgenyl β-d-glucosaminoside (**11**) with respective aldehyde, four *N*-alkyl (**37**–**40**) and six *N,N*-dialkyl derivatives (**41**–**46**) were obtained, whereas using alkylation of diosgenyl β-d-galactosaminoside (**35**) one *N*-alkyl (**47**) and two *N,N*-dialkyl (**48** and **49**) derivatives were synthesized (Figure 6).

#### 4.2.2. *N*-acyl Derivatives

**Figure 7:**
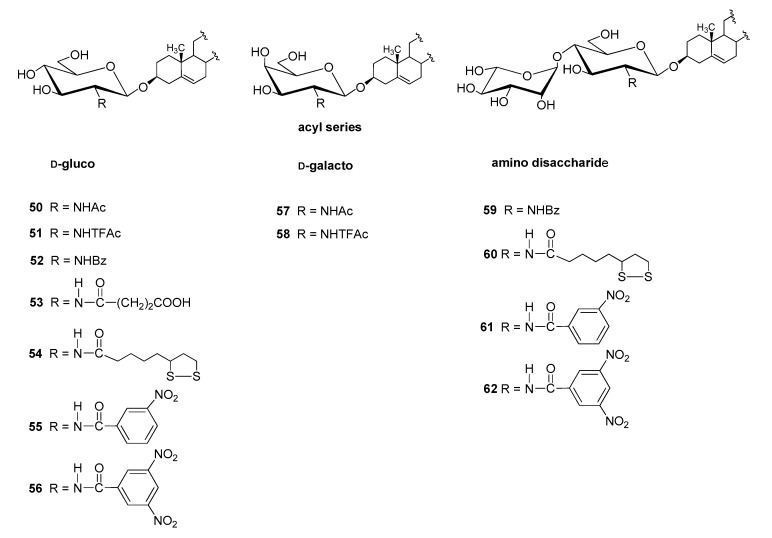
*N*-acyl derivatives of diosgenyl d-gluco- (**50**–**56**), d-galactosaminosides (**57**, **58**) and amino disaccharide (**59**–**62**).

*N*-Acetylation with acetic or trifluoroacetic anhydride, in methanol or pyridine, of the free amino group in diosgenyl β-d-glycosaminosides (**11** and **35**), provided four new saponins (**50**, **51**, **57**, **58**, Figure 7) [41,54]. 

Kaskiw at al. synthesised the other group of diosgenyl β-d-glucosaminosides with different acyl substituents at the amino group. Glycoside **36** was used for the synthesis, and the amino function of this saponin was acetylated with: benzoyl chloride, succinic anhydride in pyridine and (±)-α-lipoic acid, 3-nitrobenzoic acid, 3,5-dinitrobenzoic acid in the presence of 2-(1*H*-benzotriazole-1-yl)-1,1,3,3-tetra-methylaminium hexafluorophosphate (HBTU) and *N,N*-diisopropylethylamine (DIPEA). The final removal of the *O*-acetyl groups from obtained derivatives with sodium methoxide in CH_2_Cl_2_/MeOH mixture generated the respective diosgenyl glycosides **52*–*56** with good yields (73–92%; Figure 7) [46,47].

The same approach was used to obtain *N-*acyl derivatives of diosgenyl glycoside containing a disaccharide residue (**59**–**62**, Figure 7). After removing the Troc protecting group from the saponin **23** by treating it with zinc dust in acetic acid, the free amino group was acylated under the same conditions used for monosaccharide saponins with benzoyl chloride, (±)-α-lipoic acid, 3-nitrobenzoic acid and 3,5-dinitrobenzoic acid (76–83%). Finally, *O*-acetyl and *O*-benzoyl groups from the obtained saponins were removed by treating with sodium methoxide in methanol to yield *N-*acyl diosgenyl disaccharide consisting of glucosaminose (**59**–**62**).

**Scheme 6:**
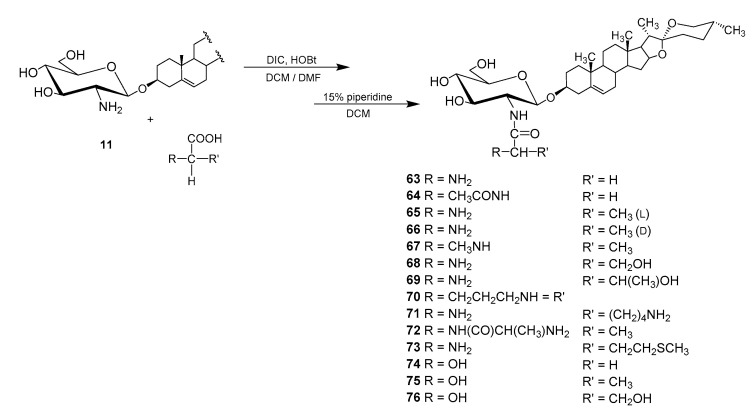
Synthesis of *N*-aminoacyl (**63**–**73**) and *N*-hydroxyacyl (**74**–**76**) derivatives of diosgenyl 2-amino-2-deoxy-β-d-glucopyranoside.

To search for pharmaceuticals that may be well-delivered to a cell, Grzywacz et al. synthesised the *N*-acyl derivatives of diosgenyl β-d-glucosaminoside (**11**) with a series of amino acids, peptide and hydroxy acids conjugated with saponin (**63**–**76**, Scheme 6) [62]. The use of amino acids and peptide as drug delivery vectors is growing due to their large structural diversity, biocompatibility as well as low toxicity [63,64]. Hydroxy acids used in these explorations are structural analogs of some amino acids. 

*N*-Aminoacyl and *N*-hydroxyacyl derivatives of diosgenyl 2-amino-2-deoxy-β-d-glucopyranoside (**63**–**76**) were obtained using the solution-phase method of peptide synthesis in DCM/DMF mixture. Fmoc-protected amino acids were used for the synthesis, whereas *N,N*’-diisopropylcarbodiimide (DIC) and 1-hydroxybenzotriazole (HOBt) were used as the coupling agents (Scheme 6). Reactions with amino acids and peptide were carried out with and without microwave assistance. In both procedures, the reaction yields were similar, but the duration was markedly reduced (even several fold) in the case of the reactions carried out in microwave reactor. Reactions of hydroxy acids were conducted solely without microwave assistance. For modifications of diosgenyl β-d-glucosaminoside (**11**), the following protected amino acids were selected: glycine, *N*-acetylglycine, sarcosine, l- and d-alanine, L-serine, l-threonine, l-lysine, l-proline, l-methionine and dipeptide (l-Ala-l-Ala). From the obtained diosgenyl *N*-aminoacyl saponins, the Fmoc-protecting group was removed by treatment with a freshly prepared 15% piperidine solution in DCM. Three hydroxy acids, namely glycolic acid, l-lactic acid and l-glyceric acid, were also attached to diosgenyl β-d-glucosaminoside (**11**). The formation of *N*-acyl derivatives of diosgenyl glucosaminoside in the presence of DIC and HOBt from the mentioned amino acids proceeds according to the known mechanism of amide bond formation using HOBt in order to minimize the formation of unreactive *N*-acylurea [65]. 

#### 4.2.3. Other Derivatives

Urea derivatives—not only of carbohydrates—exhibit a broad spectrum of biological activity. They can be part of antibiotics, enzyme inhibitors or compounds with documented cytotoxicity, such as streptozotocin or chlorozotocin [66,67]. The presence of amino group in diosgenyl glycosaminosides creates the possibility of ureido derivatives’ formation. Therefore, such saponins were also synthesised and explored.

**Figure 8:**
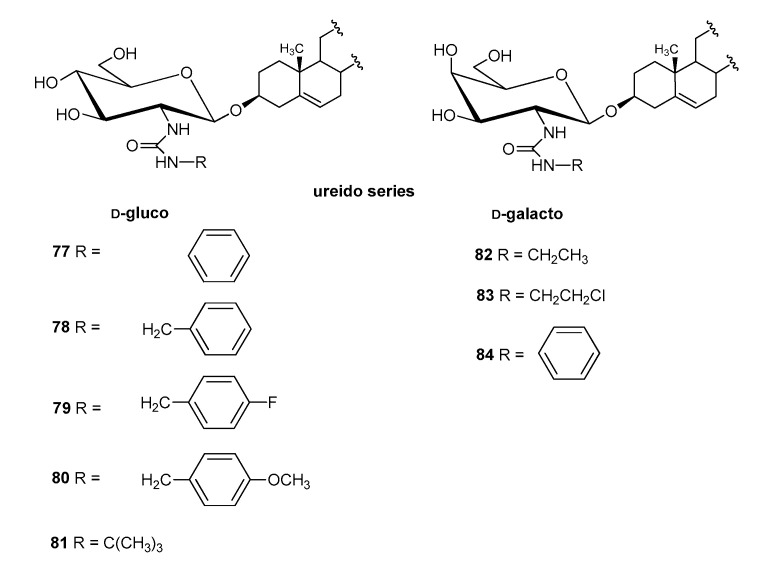
2-Ureido derivatives of diosgenyl d-gluco-(**77**–**81**) and d-galactosaminosides (**82**–**84**).

The first saponin with a 2-phenylureido moiety, glycoside **77**, was obtained by Myszka et al. in the reaction of diosgenyl β-d-glucosaminoside (**11**) with phenyl isocyanate in a CHCl_3_/MeOH mixture [34]. In the same way, using isocyanates, three different ureido derivatives of diosgenyl β-d-galactosaminoside (**82**–**84**) were obtained [54]. Wang et al. proposed a different route for the synthesis of 2-ureido diosgenyl saponins [56]. To obtain several urea derivatives (**78**–**81**) they used diosgenyl 3,4,6-tri-*O*-acetyl-*N*-Troc-2-amino-2-deoxy-β-d-glucopyranoside (**18**) and commercially available amines, including benzyl-, 4-fluoro-benzyl-, 4-methoxy-benzyl-, and *tert*-butyl-amine (Figure 8). Notably, this modification was made without the need for removal of the Troc- and *O*-acetyl-protecting group from glycoside **18**. The reactions were carried out in dimethyl sulfoxide (DMSO) at 70 °C in the presence of DIPEA. Only after completion of the reaction were the acetyl-protecting groups removed with a solution of NH_3_ in methanol. The reactions yields were 54–75%.

**Figure 9:**
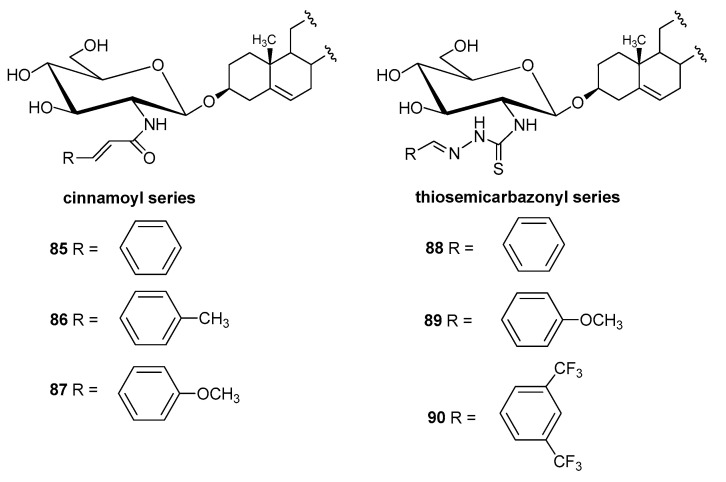
Examples of diosgenyl β-d-glucosaminosides with *N*-cinnamoyl (**85**–**87**) and 2-thiosemicarbazonyl (**88**–**90**) functional groups.

The same group of researchers synthesised two other series of diosgenyl β-d-glucosaminoside derivatives with a cinnamoyl and thiosemicarbazonyl moiety at the C-2 atom (Figure 9) [56]. Before the introduction of sulfonamides, cinnamic acid and its derivatives performed the function of chemotherapeutics. Derivatives that contained a thiosemicarbazonyl moiety can be valuable bioeffectors with a wide range of pharmaceutical applications. Some of them are antiviral, antibacterial or antifungal drugs [68,69,70].

For the synthesis of both series, it was necessary to remove the Troc protecting group (zinc dust in acetic acid) from the amine function in **18** and obtain saponin **36** (Scheme 5). The reaction of **36** with cinnamoyl chlorides with differently substituted phenyl rings formed the respective *N*-cinnamoyl derivatives of the *O*-acetylated diosgenyl glucosaminoside. Their *O*-deprotection with NH_3_ in MeOH provided a series of new diosgenyl *N*-cinnamoyl-β-d-glucosaminosides (**85**–**87**, Figure 9). Reaction yields were 55–71%.

To synthesise thiosemicarbazonyl derivatives of diosgenyl glucosaminoside (**88**–**90**), Wang et al. first converted the free amino group of **36** to isosulfocyanide in reaction of **36** with thiocarbonyl chloride in the presence of CaCO_3_ in DCM/H_2_O mixture [56]. They then treated it with 80% hydrazine hydrate in ethanol, followed by the appropriate benzaldehyde. Finally, the *O*-acetyl groups were removed, which gave saponins **88**–**90** (total yields were 48–53%).

### 4.3. Pharmacological Properties

Over the past 30 years, a significant increase in the incidence of fungal infections, multi-drug resistance to bacteria and the number of cases of cancer have been observed. Therefore, alternative therapeutic methods are being sought, in particular, effective therapies with new mechanisms of action against resistant pathogens.

Natural diosgenyl glycosides exhibit a broad spectrum of pharmaceutical properties [27,28,29,30,31,32]. The diverse biological activity of these compounds is closely connected with their diverse structural construction. Therefore, their synthetic analogs are designed and synthesised to find pharmaceutics with improved activities. This also applies to diosgenyl β-d-glycosaminosides, for which syntheses are presented above. 

#### 4.3.1. Antibacterial Activity

Saponins exhibit antibacterial activity by inhibiting the growth of Gram-positive (G+) or Gram-negative (G−) bacteria. However, some of them are not effective against G− bacteria, probably because they cannot penetrate the cell membranes of these microorganisms [71]. The cell wall of G‒ bacteria has a much more complex structure; it is composed of an additional outer membrane, phospholipids and lipopolysaccharide, which are not present in the cell wall of G+ bacteria. The mechanism of saponins’ action is based on their ability to form complexes with the sterols present in the surface membrane of eukaryotic cells/microorganisms (bacteria and fungi). As a consequence, it causes disorders of membrane integration, its perforation and rupture, and loss of intracellular components. Nucleic acids and proteins are key components of pathogens, and the integrity of the cytoplasmic membrane is essential for cell growth [72,73,74]. Studies on the obtained diosgenyl β-d-glycosaminosides indicate that none of the above-presented saponins exhibit activity against G− bacteria; the minimum inhibitory concentrations (MIC; µg/mL) were over 512 µg/mL [50,54,62].

The bactericidal effect on a large number of Gram-positive cocci were performed, setting MIC_50_, MIC_90_, and the minimum bactericidal concentration (MBC_50_ and MBC_90_; µg/mL) for diosgenyl β-d-glucosaminoside hydrochloride (**11^.^**HCl) **[75]**. The studies were conducted for clinical isolates of: methicillin- and vancomycin-resistant strains (MR and VR), as well as methicillin- and vancomycin-sensitive strains (MS and VS) of *S. aureus* and *E. faecalis*, and for *S. pyogenes* and *R. equi*. As control compounds, widely available antibiotics, including imipenem, doxycycline, erythromycin and ciprofloxacin were used (Table 2). The results of studies showed that **11^.^**HCl is equally and, in many cases, more active against examined strains to the tested antibiotics used to treat patients infected with these bacteria. Interestingly, the values of the MBC against *R. equi* for **11^.^**HCl (MBC = 4 μg/mL (50%), 16 μg/mL (90%)) were much lower than the MBC for the standard antibiotics. Additionally, when comparing the MIC_90_ and MBC_90_ values for **11^.^**HCl and for erythromycin, it can be seen that saponin **11**^.^HCl in each case gives better results, with the exception of *R. equi* bacteria (MIC_90_ = 4 μg/mL).

Further studies assessing antibacterial activity for **11**^.^HCl showed that its exhibits relative activity against tested reference strains of G+ bacteria: *E. faecalis*, *S. aureus, S. epidermidis* and *R. equi* (MIC = 16 µg/mL), whereas analogous hydrochloride of diosgenyl β-d-galactosaminoside (**35**^.^HCl) is completely inactive against the listed strains [50,54].

Additionally, the synergism of diosgenyl β-d-glucosaminoside hydrochloride (**11**^.^HCl) and antibiotics was studied [75]. The results were presented as a Fractionated Inhibitory Concentration (FIC index; FIC ≤ 0.5 indicates synergism, 0.5 < FIC ≤ 1.0 additive activity, 1.0–4 neutral effect and FIC > 4 shows an antagonistic effect). It was observed that the use of **11**^.^HCl with vancomycin or with daptomycin leads to a synergistic effect against methicillin- and vancomycin-sensitive strains, and against *R. equi* and *S. pyogenes*. The FIC index for these bacterial strains ranged from 0.312 to 0.458, whereas, for other antibiotics, the FIC value was 0.917–2.0. In no case was an antagonistic effect observed.

Taking into account the obtained synergistic results, in vivo studies on an albino strain of inbred BALB/c mice were conducted [75]. As reference antibiotics, vancomycin and daptomycin were chosen and tests were performed on infected mice with MS *S. aureus* and vs. *E. faecalis*. In the case of these infections, the number of colony-forming microorganisms, called CFU/mL, was determined. For staphylococcus-infected tissue not treated with any compound, the CFU/ml value was 6.7×10^7^ and was significantly higher than when the infected tissue was treated only with hydrochloride of **11** (CFU/ml = 4.4 × 10^4^), daptomycin (CFU/ml = 3.8 × 10^3^), or vancomycin (CFU/ml = 4.0 × 10^3^). However, when this tissue was treated with saponin **11**^.^HCl (1 mg of compound/kg of mass) plus daptomycin or vancomycin (7 mg antibiotic/kg of mass), CFU/ml values were 17 and 22, respectively, lower than for antibiotics alone. Thus, the highest bacterial inhibition was obtained for a staphylococcus-infected tissue which was treated with a mixture of saponin and antibiotic. Very similar results were obtained for vs. *E. faecalis*-infected tissue.

In turn, almost all tested mono- and dialkyl diosgenyl glycosaminosides inhibit the growth of G+ bacteria. Saponins with the *N*-ethyl group at the amino function of both d-gluco-(**37**) and d-galactosamine (**47**) are the most active compounds against reference strains of *E. faecalis*, *S. aureus, S. epidermidis* and *R. equi* (MIC = 0.5–8 μg/mL) [50,54]. Importantly, the introduction of an additional ethyl group reduces the antimicrobial activity of these derivatives, in particular with respect to *E. faecium* and *S. aureus* (for *N,N*-diethyl diosgenyl galactosaminoside **48** MIC > 1024 µg/mL). Studies have shown that several *N*-alkyl derivatives of **11** exhibit stronger or similar activity than **11**^.^HCl: saponin **38** with the *N*-propyl group (MIC = 1–8 μg/mL) and saponins **43**–**45** with *N*,*N*-dialkyl chains. In turn, two tested saponins with the longest carbons chain, *N*-pentyl (**39**) and *N,N*-diheksyl (**46**) turned out to be completely inactive against to the tested strains of G+ bacteria. These indicate that the extension of the alkyl chain, as well as the addition of another alkyl group, are rather unfavourable from the point of view of antibacterial activity. This is probably related to the lower solubility of saponins with longer alkyl chains or to the ability to form micellar structures [50].

The good activity of the *N*-acetyl derivative of diosgenyl β-d-galactosaminoside (**57**, MIC = 8–32 µg/mL for all tested reference strains of G+ bacteria) is surprising, while the analogous *N*-acetyl derivative of diosgenyl β-d-glucosaminoside (**50**) does not exhibit any microbial activity (MIC > 1024 µg/mL). In the case of *N*-aminoacyl derivatives of **11**, the dependence of saponin structure on its activity is noticeable, and only *N*-aminoacyl analogs with the free α-amino group in the aminoacyl residue are found to be active against tested reference strains of G+ bacteria [62]. Some of them exhibit better antibacterial activity than **11**^.^HCl: *N*-sarcosyl (**64**), *N*-d-and L-alanyl derivatives (**65**,**66,** respectively). Any increase or decrease in the amino acid substituent has an unfavourable effect on the biological activity of the compound, and replacing the amino group in amino acid residue by the hydroxyl group also causes a complete lack of the antibacterial activity.

#### 4.3.2. Antifungal Activity

Strains of *Candida albicans* constitute about 60% of the strains isolated from patients suffering from candidiasis, but recent data show the increasing occurrence of strains called non*-albicans Candida*. Species belonging to this group are often characterised by reduced susceptibility to antifungal agents [76].

Based on the conducted tests, the MIC values were determined for diosgenyl glycosides **11**^.^HCl, **35**^.^HCl and some of their derivatives (**37**–**49**, **63**–**76**). Hydrochloride of **11** and *N,N*-dialkyl analogs with short carbon chains (**41**–**43**), are characterised by the highest antifungal activity against reference strains *C. albicans* and *C. tropicalis* [50]. MIC values for tested fungal pathogens are in the range of 0.5–2 µg/mL. A change in the configuration of the C-4 carbon atom in the carbohydrate residue adversely affected the activity of the diosgenyl galactosaminoside. Hydrochloride of **35** in comparison with its d-gluco counterpart (**11**^.^HCl) inhibits fungal growth to a lesser extent [54]. In the case of *N*-alkyl derivatives of diosgenyl glysocaminosides, good results against both types of *Candida* were obtained for diosgenyl β-D-galactosaminosides **47**–**49** with MIC values in the range of 2–8 μg/mL, which is similar to those obtained for analogous *N*-alkyl derivatives of diosgenyl β-d-glucosaminosides (**42**,**43**). In turn, *Aspergillus niger* turned out to be the least susceptible and the majority of *N*-alkyl derivatives of diosgenyl glucosaminosides are inactive against it, with the exception of *N*-ethyl (**37**) and *N*-propyl (**38**) derivatives; MIC = 8 µg/mL. Surprisingly, *N*-acetyl analog of diosgenyl β-d-galactosaminoside (**57**) inhibits the growth of fungal reference strains, which is opposite to the corresponding d-gluco derivative (**50**).

In the case of *N*-acyl analogs, only *N*-aminoacyl derivatives of **11** with the free α-amino group in the amino acid residue are found to be active against reference strains of human pathogenic fungi (MIC = 2–4 µg/mL) [62]. Replacing this amino group by the hydroxyl function causes the lack of antifungal activities. Although part of the tested *N*-aminoacyl derivatives of **11** (**65**–**69**) inhibit the growth of *C. albicans* and *C. lypolitica*, their activity is slighter weaker than the activity of reference **11**^.^HCl. Noteworthy, for the tested *N*-acyl analogs the antifungal activity does not depend on the type of the attached amino acid, and usually the MIC values are 4 µg/mL.

In view of a large number of non*-albicans Candida* and their drug resistance, the tests of five derivatives of diosgenyl glucosaminoside (**11**^.^HCl, **41**–**44**, Figure 6) on clinical isolates were attempted [77]. These compounds were selected because of their promising MIC values against the reference strains of fungi *Candida* species. The tests were carried out on clinical strains: *C. glabrata*, *C. krusei*, *C. parapsilosis* and *C. tropicalis*, which were collected from patients suffering from vaginal, skin and mouth mycoses. As reference compounds, generally available conventional antifungal agents, including antibiotics, were used: amphotericin B, clotrimazole, fluconazole, itraconazole, natamycin and nystatin. The results of the tests are presented in Table 3.

Tested derivatives of diosgenyl glucosaminoside **11^.^**HCl, **41** and **42** exhibited a high efficacy against *C. glabrata* and *C. parapsilosis* species. They inhibit fungal growth in 90% at a concentration of 4 μg/mL and lower. Among them the most active is derivative **41**, i.e., *N,N*-dimethyl derivative, which inhibits 90% growth of *C. parapsilosis* isolates at a concentration of 2 μg/mL. These results are comparable or even stronger than those of conventional antifungal agents, such as clotrimazole and the other three antibiotics, classified as polyenes.

Two species, *C. krusei* and *C. tropicalis*, showed a certain discrepancy in their sensitivity to the tested saponins. While the MIC_50_ values for most isolates are 4–128 μg/mL, the highest concentration (1024 μg/mL) is required to inhibit the growth of individual strains in 90%. Against these two strains, three antibiotics and most conventional antifungal agents appear to be more effective. It has to be added that *C. tropicalis* species, in this examination, are characterised by significant resistance to fluconazole and itraconazole.

#### 4.3.3. Anti-Cancer Activity

Antiproliferative activity is an important biological property of natural saponins. This activity may result from programmed cell death (apoptosis or autophagy) or nonapoptotic (necrosis) and also applies to cancer cells. It has been shown that saponins have significant potential as anti-cancer agents [78].

Hydrochloride of diosgenyl β-d-glucosaminoside (**11**^.^HCl) was the first diosgenyl glycosaminoside which has been tested for cytotoxic activity. Importantly, this compound does not exhibit antiproliferative activity against non-tumoral cells—peripheral lymphocyte blood cells [48]. Further antitumor tests on **11**^.^HCl determined its independent effect and in combination with 2-chlorodeoxyadenosine (2-CdA, cladribine) on lymphocytes isolated from the patients suffering from chronic B-cell lymphocytic leukemia (B-CLL) [41]. It was found that this saponin is cytotoxic towards B-CLL—it induces apoptosis and necrosis of some leukemic B-cells. Additionally, **11**^.^HCl enhances the cytostatic effect of 2-CdA, significantly reducing (20–30%) the number of lymphatic cancer cells in some patients. This could indicate that the tested saponin increases B-cell membrane permeability and facilitates the penetration of the drug into the tumor cell. In turn, in in vitro studies on the other tumor cells, including cervical carcinoma cells—HeLa, CaSKi, ViBo—and human leukemia cells—HEL, K562, HL60 and melanoma WM9—were conducted. The hydrochloride of **11** shows only a moderate antiproliferative effect (IC_50_ ranging from 10.7 to 41 µM) [48,49]. However, it is worth adding that **11**^.^HCl shows better inhibitory activity toward the tested cancer cell lines than the starting material, which is diosgenin (IC_50_ values 63.8–81 µM) [49].

*N*-Acyl derivatives of diosgenyl glucosaminosides **50**, **52**–**56, 59**–**62** have been also examined for cytotoxic activity [46,47]. Most of the tested saponins show moderate activity against several human cancer cell lines (including SK-N-SH, MCF-7 and HeLa lines). Compound **54,** containing α-lipoic acid residue, turned out to be the most active against all three cancer cell lines (IC_50_ ranging from 4.8 µM to 7.3 µM; IC_50_ is the concentration of an inhibitor where the response is reduced by half). This cytotoxicity may be related to the redox properties of α-lipoic acid, which is a biogenic antioxidant, physiologically acting as a coenzyme in the oxidative decarboxylation of α-ketonic acids. However, the effect of this substituent on cytotoxicity is definitely smaller in the case of the α-lipoic derivative of diosgenyl amino disaccharide (**60**); the IC_50_ values for this compound increased 2-6 times in comparison to IC_50_ of **54** [47]. Further analysis of data for the other derivatives of diosgenyl amino disaccharide (**59**–**62**) confirmed that they are, in general, less active than their corresponding monosaccharides analogs with the same *N*-substitution (**52**–**56**).

In the case of *N*-cinnamoyl derivatives (**85**–**87**), SAR studies have shown that a significant effect on the cytotoxic activity clearly depends on the type of introduced substituent and its position in the benzene ring [56]. Electron-donating and electron-withdrawing substituents were introduced at various positions into the benzene ring of compound **85**. *N*-cinnamoyl derivatives with electron donating groups such as methyl (**86**) or methoxy (**87**) in the *para* position show excellent activity against HeLa and MCF-7 cell lines (IC_50_ ranging from 0.5–6.3 µM) and are much more active than the starting compound **85** (IC_50_ ranging from 12.8–39.1 µM). In turn, the introduction of such a group (OMe) in the *ortho* or *meta* position led to a significant decrease in the activity of the compound.

#### 4.3.4. Hemolytic Activity

Saponins are known to show a high ability to hemolyse red blood cells. This process causes irreversible destruction of the lipid double-layer of erythrocyte membranes and the release of hemoglobin and other intracellular components into the surrounding plasma. This may constitute a significant limitation in the use of saponins in therapies. Hemolytic activity is closely related to the structure of saponins and depends, among others, on the structure of the aglycon, on the length and number of carbohydrate units and the type of its chemical modification [17,35].

Diosgenyl β-d-glucosaminoside hydrochloride (**11**^.^HCl) and *N*-alkyl analogs (**41**–**44**) have been tested for hemolytic activity by determining the minimum hemolytic concentration (MHC) [77]. The results of tests showed that these saponins are non-toxic to human red blood cells. Hemolysis was not observed even when the erythrocytes were exposed to 256 μg/mL concentration of saponins, which is many times higher than the MIC = 2–4 μg/mL for the majority of isolated *Candida* species.

In the case of *N*-acyl derivatives of **11**, based on SAR research, it can be concluded that the ability to hemolyse red blood cells is correlated to the structure of glycosaminosides and is somewhat correlated with its antimicrobial activity [62]. *N*-Acetyl (**50**), glycyl (**63**), glycoyl (**74**) and l-lactyl (**75**) derivatives, which do not show or show very weak antimicrobial activity, also do not exhibit hemolytic activity. In turn, l-seryl (**68**), l-threonyl (**69**) and l-lysyl (**71**) derivatives of diosgenyl β-d-glucosaminoside, which exhibit better antifungal than antibacterial activity, turn out to be toxic toward red blood cells. Importantly, compounds with the highest antimicrobial activity, namely sarcosyl (**64**), l-alanyl (**65**) and d-alanyl (**66**) derivatives of **11**, are not toxic towards human red blood cells.

## 5. Conclusions

In this mini-review, various approaches to diosgenyl aminoglycosides are presented as well as various possibilities of their chemical modifications based on 2-amine function. Respective bromides, chlorides, trichloroacetimidates and (*N*-phenyl)trifluoroacetimidates are demonstrated to be useful donors for glycosidic bond formation between d-glucosamine or d-galactosamine and diosgenin. Since such a reaction demands protection of the amine function, useful protective groups are presented, such as: tetrachlorophthaloyl (TCP), trifluoroacetyl (TFA), 2,2,2-trichloroetoxycarbonyl (Troc), dimethylphosphoryl (DMP) and phthaloyl (Phth). The presented glycosidation reactions generally run with good yields, although these yields are dependent on used conditions, e.g., on the order in which the reagents are added. Regarding the modifications of the amine function, its alkylations, acylations, including acylations with amino acids and hydroxy acids, transformations into ureids and thiosemicarbazones are shown. We would like to emphasize that the presented reactions of glucosamine or galactosamine are of universal importance. The indicated protection groups and reaction pathways can be used to form a glycosidic bond between glucosamine or galactosamine and any aglycone. This universality also applies to the presented modifications of the amine function.

Presented syntheses were aimed at finding semi-natural diosgenin derivatives with favorable pharmacological properties. A wide range of such derivatives is presented, which makes it difficult to discuss the influence of specific modification on pharmacological properties. However, some conclusions can be made.

None of the tested derivatives of diosgenyl glycosaminosides are effective against G‒ bacteria. This finding confirms the known fact that saponins do not exhibit such activity.

A basic amino group is necessary for the activity of the diosgenyl glucosaminosides against G+ bacteria. Compounds with such a group, i.e., hydrochloride, some of the *N*-alkyl, *N*,*N*-dialkyl and *N*-aminoacyl derivatives, exhibit relatively strong activity against G+ bacteria. Depriving the diosgenyl glucosaminosides of the basic amino group by acetylation or replacing it with a hydroxyl group results in a loss of antibacterial activity. Among alkyl derivatives, diosgenyl *N*-ethylglucosaminoside is the most active against G+ bacteria, whereas, among *N*-aminoacyl derivatives, diosgenyl *N*-alanylglucosaminoside is the most active. In both cases, further elongations of the *N*-substituent are ineffective from the standpoint of the inhibitory activity towards the G+ bacteria. These findings are probably due to the lower solubility of the compounds with longer *N*-substituents or to the micelle formation.

Basic amino group is also necessary for the activity of the diosgenyl glucosaminosides against tested *Candida* species. The growth of tested fungi is the most efficiently inhibited by the hydrochloride of diosgenyl glucosaminoside and its alkyl derivatives with short carbon chains (*N*-ethyl and *N*,*N*-dimethyl). *N*-aminoacyl derivatives of diosgenyl glucosaminoside quite effectively acted against fungi, and this effect is independent of the size of amino acid. Again, replacing the amino group by the amido group (acylation), as well as replacing the α-amino group by the hydroxyl group, causes the antifungal activities to decrease.

Switching of the d-glucosamine into d-galactosamine dramatically changes antimicrobial properties of the respective diosgenyl glycosides. While the hydrochloride of diosgenyl glucosaminoside is active against G+ bacteria and against *Candida*, the hydrochloride of diosgenyl galactosamine is not active at all. Conversely, *N*-acetyl derivative of diosgenyl glucosaminoside does not exhibit any antibacterial nor antifungal activity, whereas its galactosamine analogue acts against G+-bacteria-tested and against *Candida*-tested. This result may suggest that these two diosgenyl glycosaminosides act according to different mechanisms.

With regard to anti-cancer properties, promising results were obtained for the hydrochloride of diosgenyl glucosaminoside and some acyl derivatives, particularly for the α-lipoic acid derivative. No alkyl nor aminoacyl derivatives of diosgenyl glycosaminosides were tested for their anti-tumor activity.

Extremely important is that derivatives of diosgenyl d-glucosaminosides, which are active against antimicrobials and/or cancers (hydrochloride, *N*-alkyl, *N*-aminoacyl active), are non-toxic to human red blood cells.

The conclusions drawn in this work may be helpful in designing further modifications of diosgenyl glycosaminosides as well as in designing modifications of other glycosaminosides aimed at the search for effective pharmaceuticals.

## Figures and Tables

**Table 1:** Most commonly used procedures for glycosylation of diosgenin.

Entry	Procedure	Glycosyl Donor			Solvent	Promotor	Product	Yield (%)	Lit.
1	*normal*	**5** (α)	Bromide	NHTFAc	CH_2_Cl_2_	AgOTf	**8**	65	[39]
2	*normal*	**5** (α)	Bromide	NHTFAc	CH_2_Cl_2_/Et_2_O	AgOTf	**8**	69	[41]
3	*reverse*	**5** (α)	Bromide	NHTFAc	CH_2_Cl_2_/Et_2_O	AgOTf	**8**	77	[50]
4	*normal*	**7** (α + β)	Bromide	NTCP	CH_2_Cl_2_	AgOTf	**9**	65	[39]
5	*normal*	**7** (α + β)	Bromide	NTCP	CH_2_Cl_2_/Et_2_O	AgOTf	**9**	73	[41]
6	*reverse*	**7** (α + β)	Bromide	NTCP	CH_2_Cl_2_	AgOTf	**9**	93	[50]
7	*normal*	**10** (α)	TCAI	NDMP	CH_2_Cl_2_	TMSOTf	**15**	92	[45]
8	*normal*	**11** (α)	TCAI	NHTroc	CH_2_Cl_2_	TMSOTf	**16**	98; 84	[47,56]
9	*normal*	**12** (α + β)	TCAI	NPhth	CH_2_Cl_2_	TMSOTf	**17**	96; 80	[48,49]
10	*reverse*	**13** (α)	Bromide	NHTroc	CH_2_Cl_2_/Et_2_O	AgOTf	**16**	98	[50]
11	*normal*	**14** (α + β)	Bromide	NPhth	CH_2_Cl_2_/Et_2_O	AgOTf	**17**	51	[50]
12	*reverse*	**14** (α + β)	Bromide	NPhth	CH_2_Cl_2_/Et_2_O	AgOTf	**17**	55	[50]
13	*reverse*	**14** (α + β)	Bromide	NPhth	CH_2_Cl_2_	AgOTf	**17**	90	[50]
14	*reverse*	**18** (α)	Chloride	NHTFAc	CH_2_Cl_2_/Et_2_O	AgOTf	**8**	69	[43]
15	*reverse*	**19** (α)	Choride	NHTroc	CH_2_Cl_2_/Et_2_O	AgOTf	**16**	86	[43]
16	*reverse*	**20** (β)	Chloride	NPhth	CH_2_Cl_2_	AgOTf	**17**	99	[43]
17	*reverse*	**21** (β)	Chloride	NTCP	CH_2_Cl_2_	AgOTf	**9**	87	[43]
18	*normal*	**22** (α + β)	PTFAI	NHTFAc	CH_2_Cl_2_	TMSOTf	**8**	85	[50]
19	*normal*	**23** (α + β)	PTFAI	NHTroc	CH_2_Cl_2_	TMSOTf	**16**	81	[50]
20	*normal*	**24** (β)	PTFAI	NPhth	CH_2_Cl_2_	TMSOTf	**17**	83	[50]
21	*normal*	**25** (β)	PTFAI	NTCP	CH_2_Cl_2_	TMSOTf	**9**	52	[50]
22	*reverse*	**27** (α + β)	Bromide	NTCP	CH_2_Cl_2_	AgOTf	**28**	80	[54]

**Table 2:** Selected MIC_50_ (MIC_90_) and MBC_50_ (MBC_90_) values for **11**^.^HCl and clinically used antibiotics against clinical isolates of Gram-positive bacteria.

	MIC_50_ (MIC_90_) *						MBC_50_ (MBC_90_) **					
	*MR S. aureus* (*n* = 20) ***	*MS S. aureus* (*n* = 20)	*VR E. faecalis* (*n* = 10)	*VS E. faecalis* (*n* = 20)	*S. pyogenes* (*n* = 20)	*R. equi* (*n* = 20)	*MR S. aureus* (*n* = 20)	*MS S. aureus* (*n* = 20)	*VR E. faecalis* (*n* = 10)	*VS E. faecalis* (*n* = 20)	*S. pyogenes* (*n* = 20)	*R. equi* (*n* = 20)
**11**^.^HCl	4 (8)	2 (4)	8 (32)	8 (16)	2 (4)	2 (4)	8 (16)	8 (16)	16 (64)	16 (32)	4 (8)	4 (16)
**Imipenem**	16 (128)	1 (2)	16 (64)	4 (16)	0,5 (1)	0,25 (1)	64 (256)	4 (8)	64 (128)	16 (64)	1 (4)	8 (32)
**Doxycycline**	4 (16)	4 (8)	16 (32)	8 (16)	4 (8)	1 (2)	8 (32)	8 (32)	16 (64)	16 (64)	8 (16)	32 (128)
**Erythromycin**	4 (16)	2 (8)	16 (64)	8 (32)	2 (8)	0,5 (2)	32 (128)	16 (64)	32 (128)	16 (128)	8 (32)	32 (64)
**Ciprofloxacin**	4 (8)	2 (8)	8 (32)	4 (8)	2 (8)	1 (2)	8 (16)	4 (16)	16 (128)	8 (16)	8 (16)	16 (64)

* MIC_50_ (MIC_90_) = minimum inhibitory concentrations (µg/mL) at which 50% and 90% of the isolates were inhibited, respectively ** MBC_50_ (MBC_90_) = minimum bactericidal concentration (µg/mL) at which 50% and 90% of the isolates were inhibited, respectively *** n is the number of tested isolates of a given bacterium.

**Table 3:** MIC_50_ * and MIC_90_ * values for hydrochloride of **11,** *N,N*-dialkyl saponins **41**–**44** and antifungal agents against clinical isolates of fungi of genus *non-albicans Candida*.

Saponin Antibiotic		*C. glabrata* (*n* = 22) **		*C. krusei* (*n* = 12)		*C. parapsilosis* (*n* = 19)		*C. tropicalis* (*n* = 13)	
	Fungus	50%	90%	50%	90%	50%	90%	50%	90%
**11**^.^HCl		2	4	16	1024	2	4	4	1024
**41**		4	4	16	1024	1	2	4	1024
**42**		4	4	64	1024	2	4	4	512
**43**		4	8	1024	1024	4	4	8	1024
**44**		8	16	1024	1024	8	16	128	1024
**Amphotericin B**		2	2	2	2	2	4	1	2
**Clotrimazole**		4	8	0.25	0.25	0.25	0.25	4	16
**Fluconazole**		128	128	32	64	4	8	128	1024
**Itrakonazol**		4	32	0.25	0.25	0.25	0.25	256	1024
**Natamycin**		2	2	1	1	4	4	2	4
**Nystatin**		8	8	2	4	4	8	2	4

* MIC_50_ (MIC_90_) = minimum inhibitory concentrations (µg/mL) at which 50% and 90% of the isolates were inhibited, respectively. ** n is the number of tested isolates of a given pathogen.
